# Intracranial Retrograde Cerebrospinal Fluid Dissemination of H3 K27-altered Glioma

**DOI:** 10.1055/a-2780-4173

**Published:** 2026-01-12

**Authors:** Parth Patel, James Battiste, Kar-Ming Fung, Ian F. Dunn, Christopher S. Graffeo

**Affiliations:** 1 Department of Neurosurgery, University of Oklahoma, Oklahoma City, Oklahoma, United States; 2 Department of Pathology, University of Oklahoma, Oklahoma City, Oklahoma, United States

**Keywords:** intracranial dissemination, diffuse midline glioma, H3K27M mutant, H3 K27-altered

## Abstract

**Introduction:**

Diffuse midline glioma, H3 K27-altered (DMGHA), is an uncommon malignant primary brain tumor associated with a grave prognosis and limited treatment options. We report a unique case of an elderly woman who was initially treated for a right cerebellar DMGHA. She subsequently developed a distal recurrence at the septum pellucidum, hypothesized to be a result of retrograde cerebrospinal fluid (CSF) dissemination.

**Case History:**

A 72-year-old woman initially presented with headache and dizziness. The subsequent workup revealed a right cerebellar mass. A diagnosis of DMGHA was confirmed by histological studies and molecular profiling, and she was treated with gross total resection followed by protocol-based chemoradiation. At 12 months after surgery and treatment, surveillance imaging showed a new isolated enhancing mass in the septum pellucidum. She underwent another gross total resection with an anterior interhemispheric approach. Pathology confirmed a diagnosis of DMGHA.

**Conclusion:**

We report a novel case of recurrent distal spread of a right cerebellar DMGHA to the septum pellucidum. Although several putative mechanisms may be hypothesized to account for the very unique pattern of disease spread including simple multifocal disease, retrograde CSF dissemination may be the most likely mechanism. This is due to the lack of radiographic evidence for subcortical or hematogenous spread, and the interface between both enhancing masses and the CSF compartment.

## Introduction

Diffuse midline glioma, H3 K27-altered, CNS WHO grade 4 (DMGHA) is an uncommon and highly aggressive primary central nervous system (CNS) tumor, first introduced in the 2016 WHO Classifications for CNS Tumors. Although more common in children, adult diagnosis of DMGHA is increasingly prevalent and similarly associated with an unfavorable prognosis—especially as compared with H3-wild type tumors. 1 DMGHA typically exhibits classical patterns of spatiotemporal evolution and disease spread that are aligned with phenotypically routine behavior for infiltrating gliomas. 2 3 4 We report a novel case of cerebellar DMGHA that recurred at the septum pellucidum, potentially a result of retrograde cerebrospinal fluid (CSF) dissemination.

## Case History

A 72-year-old woman presented to our emergency department with new-onset progressive headache and dizziness with worsening of her baseline chronic dysphagia. Her family history was notable for a twin sister diagnosed with and treated for a low-grade temporal lobe astrocytoma at 21-years-old, which ultimately led to her death at 34 years of age.

**Fig. 1:**
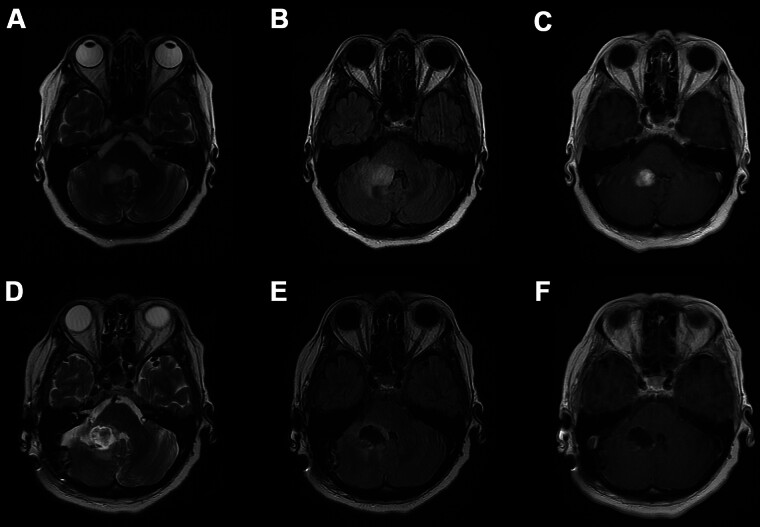
Initial ( **A** ) axial T2-weighted MRI, ( **B** ) axial T2-weighted FLAIR MRI, and ( **C** ) axial gadolinium contrast-enhanced T1-weighted MRI demonstrating presence of a cerebellar mass. Postoperative ( **D** ) axial T2-weighted MRI, ( **E** ) axial T2-weighted FLAIR MRI, and ( **F** ) axial gadolinium contrast-enhanced T1-weighted MRI demonstrating gross total resection of the cerebellar mass.

The patient presented to an outside hospital 3 months prior to presentation at our institution with severe nausea and vertigo. At that time, a mass was detected. She was treated with dexamethasone and referred to our institution for further treatment. Magnetic resonance imaging (MRI) of the brain obtained at that time revealed an enhancing posterior fossa mass involving the anteromedial aspect of the right cerebellar hemisphere. T2/FLAIR sequence demonstrated change extending into the posterolateral aspect of the right pontis brachium, with greatest measurement of 1.8 cm in the coronal plane ( Fig. 1A–C ). Neurologic examination was grossly intact, apart from mild cognitive impairment that was considered within normal limits for a 72-year-old woman with multiple chronic medical conditions.

**Fig. 2:**
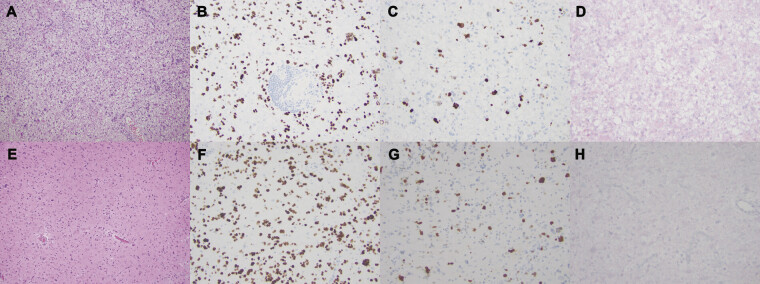
Microscopic pathologic imaging from the cerebellar mass with ( **A** ) hematoxylin and eosin stain demonstrating moderately cellular glial neoplasm with occasional mitotic activities, ( **B** ) reactive H3 K27M immunohistochemical stain, ( **C** ) Ki67 stain with labeling index around 5%, and ( **D** ) nonreactive IDH1 R132H immunohistochemical stain. Microscopic pathologic imaging from the septum pellucidum mass with ( **E** ) hematoxylin and eosin stain consistent with diffuse midline glioma, ( **F** ) reactive H3 K27M immunohistochemical stain, ( **G** ) Ki67 stain with labeling index around 15%, and ( **H** ) nonreactive IDH1 R132H immunohistochemical stain.

The patient underwent right retrosigmoid craniotomy for gross total resection of the mass, which resulted in new mild ipsilateral ataxia and moderate worsening of her baseline dysphagia. Postoperative MRI ( Fig. 1D–F ) confirmed gross total resection of the mass, while pathological analysis ( Fig. 2A–D ) confirmed DMGHA. The postoperative period was uneventful, and the patient was discharged to inpatient rehabilitation on postoperative day 4. As per current protocol recommendations, radiotherapy to a total dose of 4,000 cGy in 15 fractions and one full cycle of temozolomide was implemented. Afterwards, she was unable to tolerate additional cycles due to severe fatigue, nausea, constipation, stomach pain, weight loss, and dysphagia. She was subsequently followed with routine radiographic surveillance with no additional adjuvant therapies.

**Fig. 3:**
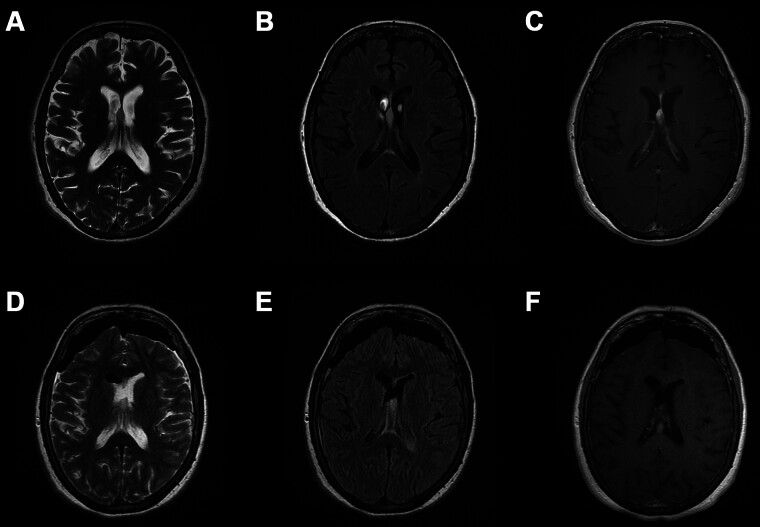
Surveillance ( **A** ) axial T2-weighted MRI, ( **B** ) axial T2-weighted FLAIR MRI, and ( **C** ) axial gadolinium contrast-enhanced T1-weighted MRI demonstrating new septum pellucidum mass. Postoperative ( **D** ) axial T2-weighted MRI, ( **E** ) axial T2-weighted FLAIR MRI, and ( **F** ) axial gadolinium contrast-enhanced T1-weighted MRI demonstrating gross total resection of the septum pellucidum mass.

For the first year, surveillance MRI was unremarkable. 12 months after resection, interval imaging revealed a new enhancing mass arising from the septum pellucidum ( Fig. 3A–C ). Of note, there was no radiographic evidence of subcortical or hematogenous spread, no evidence of local recurrence in the right cerebellum, and no other focus of new disease spread. At 14 months after the initial cerebellar operation, the patient underwent an anterior interhemispheric approach for gross total resection ( Fig. 3D–F ) of the mass. There was no major decline whatsoever, and the patient awoke with transient encephalopathy which lasted 9 days and further progression of her baseline dysphagia ultimately requiring feeding tube placement prior to discharge home on postoperative day 10. New pathology ( Fig. 2E–H ) demonstrated very little change in morphology compared with the first pathologic sample which helped establish a common final diagnosis of DMGHA (WHO Grade 4). The patient declined further adjuvant therapy because of her previous difficulties with temozolomide therapy and her overall frailty. 4 months after resection of the septal mass, the patient developed worsening bilateral leg weakness and mixed aphasia with generalized decompensation, prompting her family to pursue hospice. She ultimately died 3 months after transitioning to hospice, which was 7 months after the second resection, and 21 months after the initial cerebellar resection.

## Discussion

DMGHA was first defined as a pathological entity in the 2016 update to the WHO Classification for CNS Tumors. 5 Although limited case accumulation exists for this diagnosis among adults, adults tend to have longer overall survival compared with children due to a higher percentage of adults having more favorable histology. 6 For adults, DMGHA tends to occur more often under the age of 40, but DMGHA has been documented to occur in elderly patients. 7 8 Although cerebellar DMGHA occurs less often in adults compared with other midline locations, infratentorial DMGHA is associated with worse overall survival compared with wildtype tumors. 6 8 9 Among adults of all ages, previous studies have reported DMGHA to have a median progression-free survival of 7 to 11 months and overall survival of 10 to 30 months which is similar to what we observed in our case. 6 10

Distant spread has been observed through a variety of mechanisms, predominantly more direct infiltration, although CSF dissemination has similarly been reported. 2 3 4 Spread of any CNS tumor via retrograde CSF dissemination is extremely rare and reported through scattered cases, none of which has expressed the H3K27M mutation. 11 To our knowledge, the present study is the index report of a presumed retrograde CSF dissemination for a DMGHA, as well as a rare instance with a more intermediate phenotype, and a relatively robust survival for an elderly patient—especially when accounting for her baseline frailty and medical complexity. In general, retrograde spread of a CNS tumor through the CSF has been described by isolated case reports; however, these are exceedingly rare, and none has described a case of DMGHA. 11 Although the spinal cord or downstream ventricles should be the most likely location for tumor recurrence via CSF dissemination, no radiographic evidence of tumor spread was present in these locations. If our hypothesis was correct and the patient had survived longer, the tumor may have spread to the spinal cord and ventricles.

Given its predilection for critical midline locations including the brainstem, basal ganglia, and thalamus, DMGHA is frequently encountered in locations that prohibit aggressive resection. Correspondingly, the standard of care is typically a needle biopsy to confirm a tissue diagnosis, followed by radiotherapy, with the addition of chemotherapy determined by the patient's performance status and other disease features. 7 In some instances, such as the present case, safe maximal resection may be surgically feasible; this is relatively uncommon, but should be the preferred strategy where possible. 12

Diffuse midline glioma is most commonly found in midline structures such as the thalamus, brainstem, or spinal cord, among both adult and pediatric cohorts. 13 This signature disease feature reflects association of DMGHA with progeniture cells associated with midline structure maturation in normal developmental pathways. 8 Many other anatomical locations have been observed for DMGHA, including less prevalent midline or paramedian locations, as seen with both disease foci in the present operation. 6 8

We report a novel case of presumed retrograde CSF dissemination in an adult with cerebellar DMGHA with spread to the septum pellucidum. Although she ultimately died from disease, her case was managed successfully for 21 months after initial diagnosis through a combination of neurosurgical, neuro-oncologic, and radiotherapeutic strategies—a relatively robust survival for an elderly woman diagnosed with a very aggressive malignant glioma variant. Taken together with the shared interface between both enhancing masses and the CSF compartment, retrograde CSF dissemination was considered the most likely mechanism underlying the distal spread of disease. Compared with other high-grade gliomas, DMGHA exhibits a peculiar dissemination pattern where distal spread is frequently observed in advance of more profound local enlargement at the primary disease site. 4 Distal recurrence may occur without marked progression at the initial location, although a pattern of infiltrating spread is far more common than multifocal disease without primary progression or recurrence. At present, two highly unusual cases of extraneural metastasis from DMGHA have been reported in adult patients; both were instances of secondary tumor growth within the bony skull after treatment of a primary midline lesion. 12 14 In children, retrograde spread has been very rarely reported, with intracranial metastasis arising in the wake of a primary spinal DMGHA via a presumed CSF dissemination route. 15 The present case represents the first such case in an adult patient, as well as the first instance of intraventricular retrograde spread, rather than spine-to-brain retrograde spread.

The present case is subject to numerous limitations, principally derived from the rarity of the DMGHA diagnosis which yields a clinical phenotype that lacks sufficient granularity in its understanding. 1 Furthermore, although the radiographic and pathologic evidence are suggestive of retrograde CSF dissemination, this pathway cannot be demonstrably proven using the conventional clinical elements at our disposal. The challenges of substantiating such a claim are perhaps best illustrated by considering the lack of CSF data from this patient; if positive, this would have supported the study hypothesis, but not proven it.

## Conclusion

We report a novel case of recurrent metachronous DMGHA spread via a presumed mechanism of retrograde CSF dissemination. A relatively rare diagnosis in elderly adults, DMGHA is rare, aggressive, and prognostically unfavorable, with characteristically rapid progression to disease fatality. This present case offers a unique perspective on a disease with emerging and evolving understanding, while also emphasizing the need for close surveillance and aggressive treatment whenever possible. Further case accumulation and pathobiological data are required to develop more meaningful insights regarding the natural history, optimal treatment, and avenues for intervention in these highly vulnerable patients.
